# How good is a living donor? Systematic review and meta-analysis of the effect of donor demographics on post kidney transplant outcomes

**DOI:** 10.1007/s40620-021-01231-7

**Published:** 2022-01-24

**Authors:** Maria Irene Bellini, Mikhail Nozdrin, Liset Pengel, Simon Knight, Vassilios Papalois

**Affiliations:** 1 Department of Surgical Sciences, Sapienza University of Rome, 00161 Rome, Italy; 2 Imperial College School of Medicine, London, SW7 2AZ UK; 3 Centre for Evidence in Transplantation, Nuffield Department of Surgical Sciences, University of Oxford, Oxford, OX3 7HE UK; 4 Department of Surgery and Cancer, Imperial College, London, SW7 2AZ UK

**Keywords:** Living donation, Donor’s demographics, Graft outcomes, Kidney transplantation

## Abstract

**Background and Aims:**

Living donor kidneys are considered the best quality organs. In the attempt to expand the donor pool, the donor’s age, sex and body mass index (BMI) might be considered as potential determinants of the kidney transplant outcomes, and thus guide recipient selection. We aimed to investigate the effects of donor demographics on kidney function, graft and recipient survival, delayed graft function (DGF) and acute rejection (AR).

**Methods:**

Systematic review and meta-analysis. EMBASE, MEDLINE, Web of Science, BIOSIS, CABI, SciELO and Cochrane were searched using algorithms. NHBLI tools were used for risk of bias assessment. Mean difference (MD), standardized mean difference (SMD), and risk ratio (RR) were calculated in Revman 5.4

**Results:**

Altogether, 5129 studies were identified by the search algorithm; 47 studies met the inclusion criteria and were analyzed. No significant difference in recipient 1-year survival was found between recipients of donors aged < 50 vs donors aged > 50 (RR = 0.65 95% CI: 0.1–4.1), and recipients of donors aged < 60 vs donors aged > 60 (RR = 0.81 95% CI: 0.3–2.3). Graft survival was significantly higher in recipients of grafts from donors aged < 60. Risk of AR (RR = 0.62 95% CI: 0.5–0.8) and DGF (RR = 0.28 95% CI: 0.1–0.9) were significantly lower in recipients of grafts from donors aged < 60. One-year serum creatinine was significantly lower in recipients from donors aged < 60 years compared to donors aged > 60 years (MD = 0.3 mg/dl 95% CI: 0.1–0.9), although there was high heterogeneity. Recipients of grafts from male donors had lower 1-year serum creatinine (MD = 0.12 mg/dl 95% CI: 0.2–0.1) and higher eGFR compared to recipients of female donors (p < 0.00001). Donor obesity increased the incidence of delayed graft function but not acute rejection (RR = 0.66 95% CI: 0.32–1.34).

**Conclusions:**

Older donor age was associated with worse post-transplant outcomes and recipients of male donors had better 1-year eGFR. Donor obesity affects the incidence of delayed graft function, but not the incidence of acute rejection in recipients.

**Graphical Abstract:**

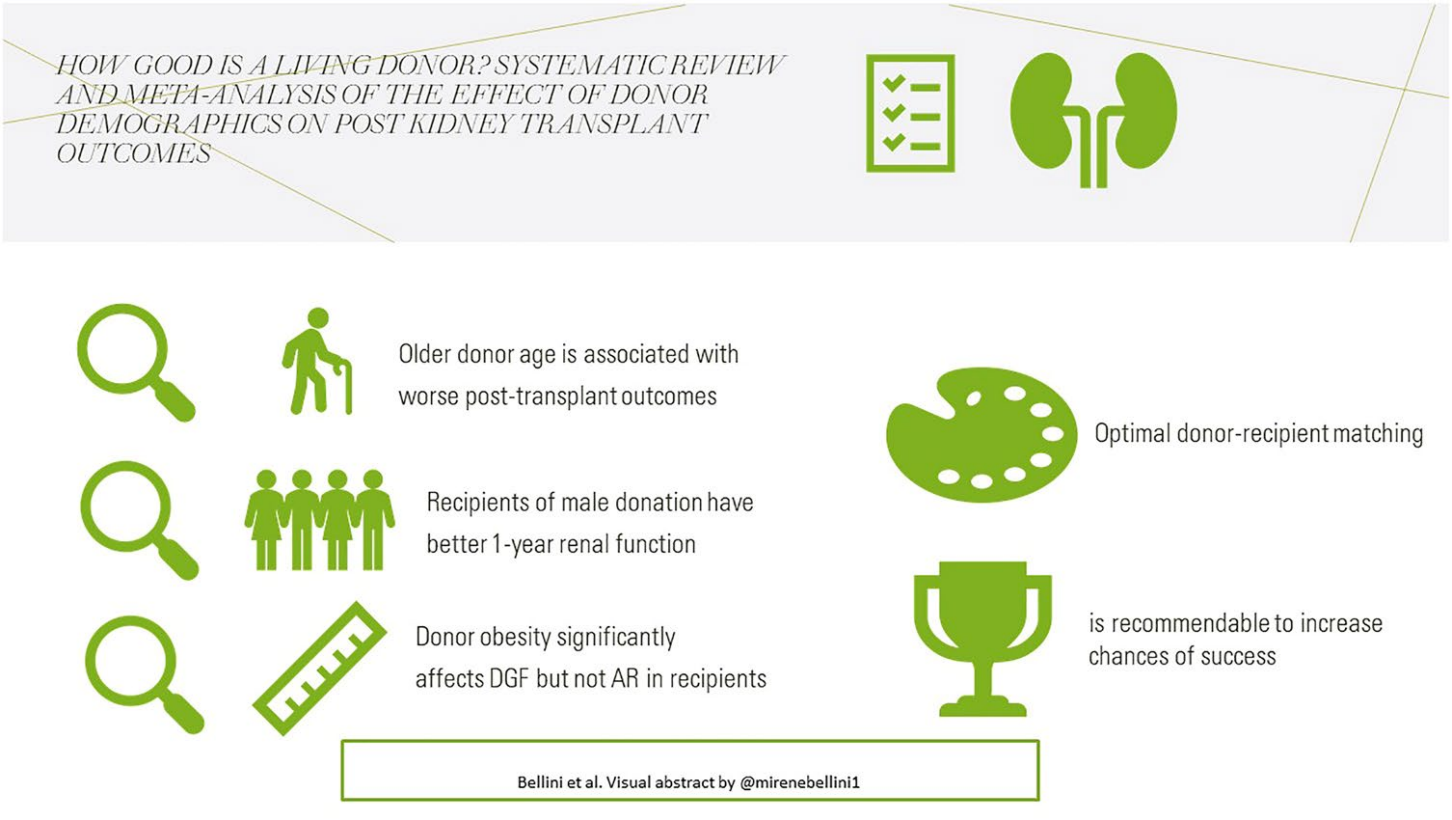

**Supplementary Information:**

The online version contains supplementary material available at 10.1007/s40620-021-01231-7.

## Introduction

Long-lasting results after kidney transplantation are largely influenced by the quality of the organ received, with living kidney donation (LKD) offering the best transplant outcomes [1].

To manage the current organ donor shortage, the transplant community has progressively opened up to a broadening of the selection criteria for living donor candidates, in terms of donor age and body mass index (BMI), with no definitive cut-off being accepted [2].

In the case of deceased donation, strategies to improve outcomes consequent to the acceptance of extended criteria donors, namely a higher incidence of delayed graft function (DGF), [3] are currently under investigation, in consideration of the higher impact of an ischemic-reperfusion injury in these organs [4]. Yet, a comparable evaluation of extended criteria for living donors is missing, in particular with regard to the donor’s demographic characteristics of sex, age and BMI.

It has been previously reported that a higher proportion of wife-to-husband donations and disproportionate female-to-male donations among biological relatives and unrelated pairs, lead to gender inequality in kidney transplantation [5, 6]. The same inequity remains underrepresented in many clinical research studies, thus limiting the evidence based upon which to make recommendations to ensure the best outcomes.

With regard to BMI, controversy still exists, with some advocating bariatric surgery as a pre-donation procedure [7], and others excluding candidates who do not fit the center’s criteria [8]. We previously investigated the effects of the recipients’ demographic characteristics on outcomes of kidney grafts from living donors (LDs) [9]; the aim of the present study is to investigate the effects of LKD demographics on kidney graft function and survival.

## Methods

**Fig. 1:**
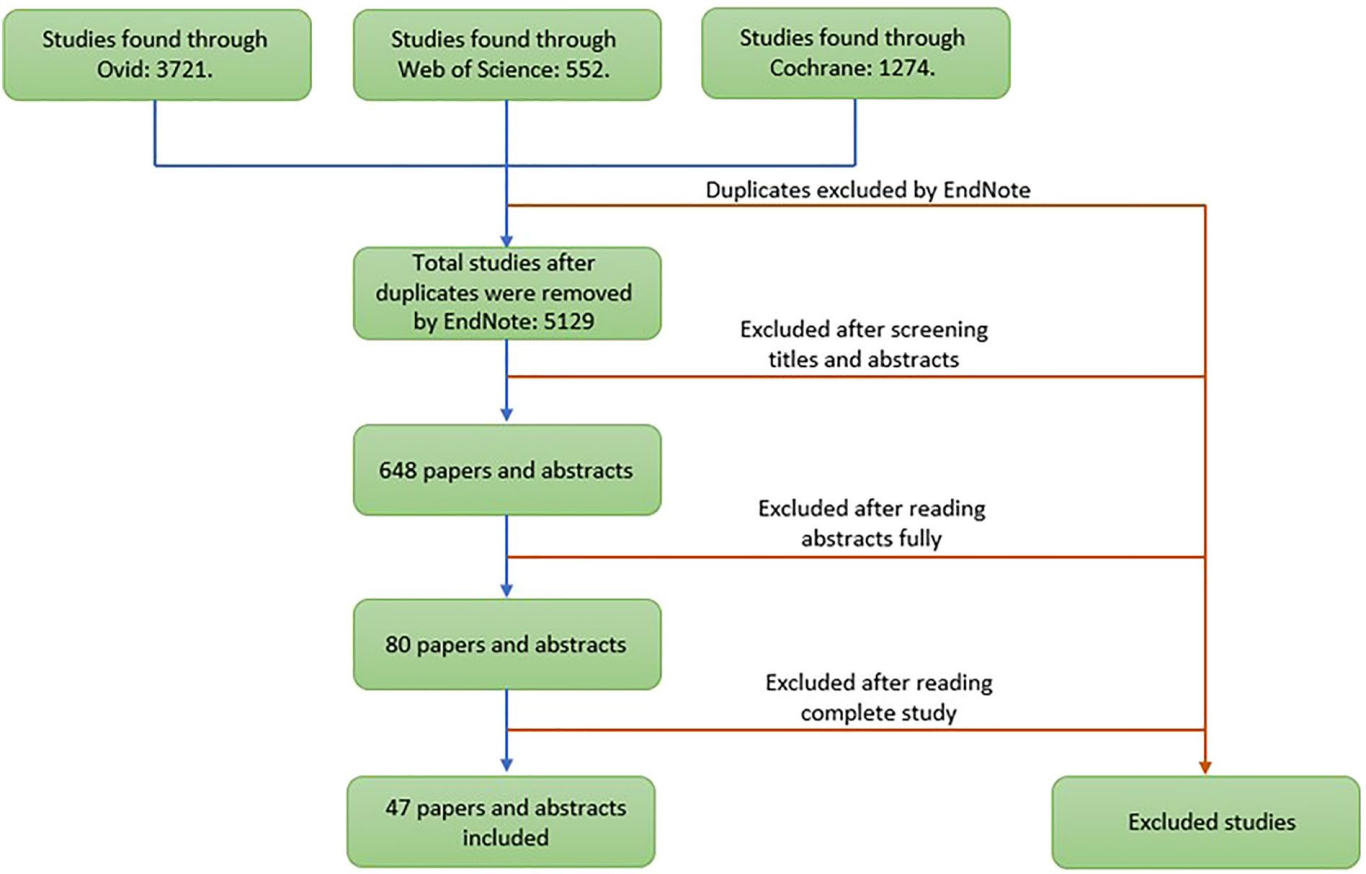
PRISMA flowchart

The review was conducted and reported according to PRISMA guidelines [10], Fig. 1, and MOOSE criteria [11].

### Search strategy

Literature searches were performed in Ovid (EMBASE, MEDLINE), Web of Science and Cochrane databases, using combinations of free text and keyword terms for living kidney donation and donor demographics of interest. Searches were conducted on the 14/11/20 and are reported in Appendix 1 in ESM.

### Inclusion/exclusion criteria

Any study relating living kidney donor demographics to recipient outcomes were eligible for inclusion, including full articles and meeting abstracts. Only studies in English were included for the analysis.

### Outcomes of interest

The effect of donor demographics of age, sex, BMI, and genetic relationship to the recipient on patient survival and graft function evaluated using estimated glomerular filtration rate (eGFR) adjusted for body surface area and serum creatinine, proteinuria incidence, delayed graft function and acute rejection (AR) were investigated.

### Screening and data extraction

Study identification and data extraction were performed in three stages: the first stage included downloading the studies identified by the search strategy from Cochrane, Ovid and Web of Science databases into EndNote reference management software. The reference management software was then used to remove duplicate studies. The second stage included two independent researchers (MIB and MN) screening the titles and abstracts of long-listed studies. The researchers then each produced a list of studies they thought would be eligible for the review. The two lists were then compared to see whether one of the reviewers excluded a potentially viable study. A single short-list of studies selected for full text review was then produced. The third stage of data extraction included the researchers fully reading the short-listed studies and identifying the studies that met the inclusion criteria. Data extraction was performed by two independent reviewers (MIB and MN) and disagreements were solved by discussion or consulting a third reviewer. Data was extracted into a Microsoft Excel sheet.

### Risk of bias assessment

Risk of bias assessment (Appendix 2 in ESM) was performed using the National Institute of Health National Heart, Lung and Blood Institute (NIH NHBLI) quality assessment tool [12]. Two independent reviewers, MIB and MN, judged the quality of the articles and compared their results. Risk of bias assessment was not carried out for congress abstracts included in the study (4 abstracts).

### Meta-analysis

All data analyses were performed in Revman 5.4.1 and IBM SPSS Statistics 26. Meta-analysis of mean difference was used for continuous data. Random effect models were used for all meta-analyses due to the heterogeneous and small study samples. Mean differences with a 95% confidence interval were calculated for the summary effect. The Z test was performed to calculate p-values. Where p-values were < 0.05 and 95% CI did not include 0, a statistically significant difference between the two groups was recorded. Forest plots were created in Revman 5.4.1.

When it was necessary to combine two reported subgroups into a single group for the meta-analysis (for example combining subgroups of donors aged 18–24 with donors aged 24–50 into a single group to compare it against a group of donors over the age of 50), the formula for combining groups from the Cochrane handbook was used [13].

## Results

### Effect of donor age

**Fig. 2:**
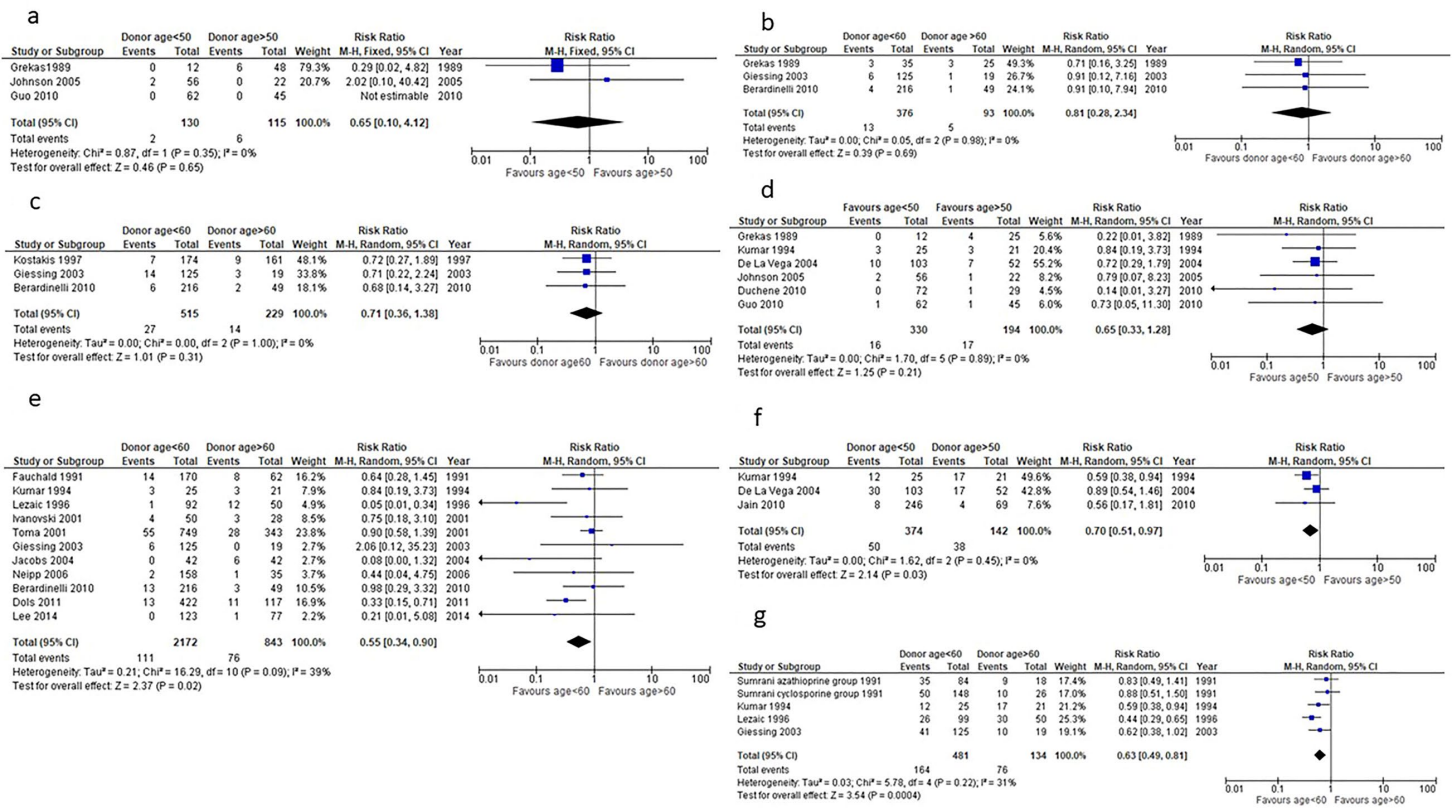

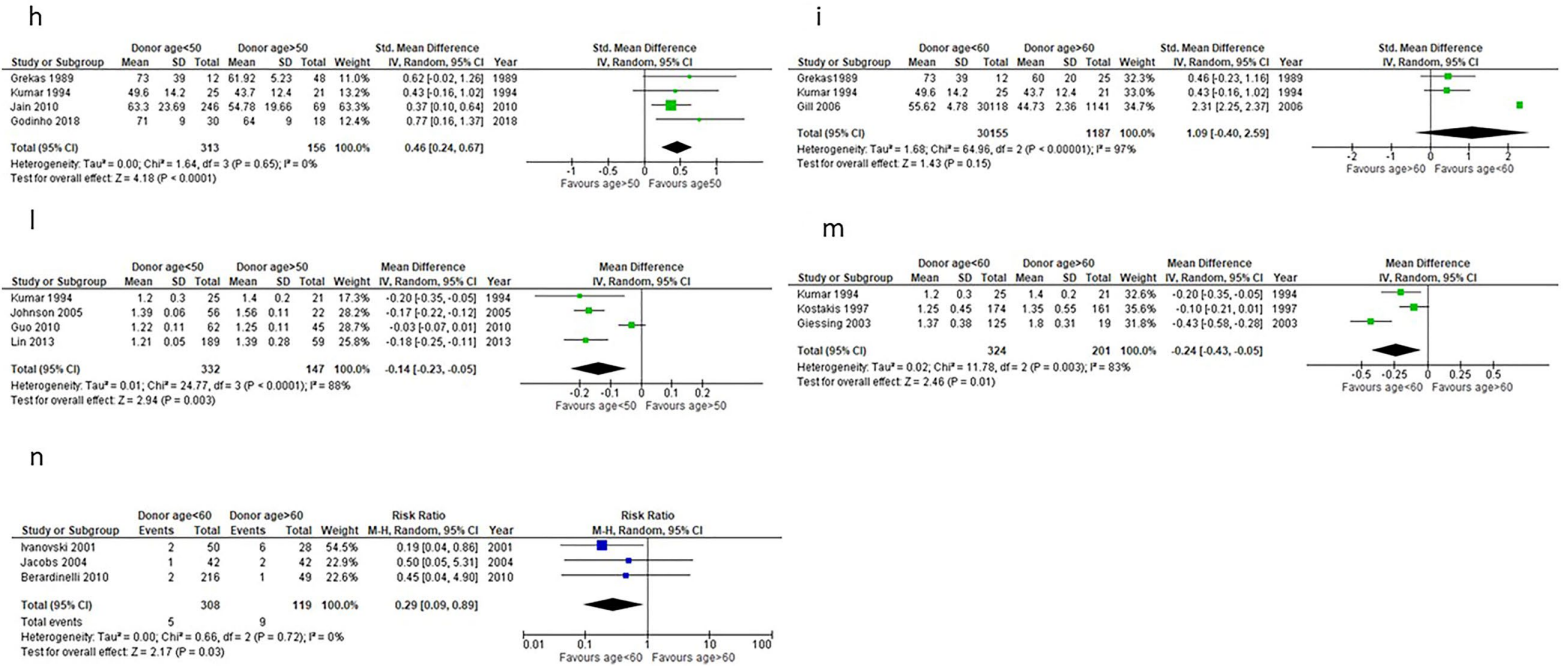
**a** Comparison of 1-year recipient death between renal transplant recipients from donors aged less than 50 years and donors older than 50 years. **b** Comparison of 1-year graft death between renal transplant recipients from donors aged less than 60 years and donors older than 60 years. **c** Comparison of 3-year recipient survival between renal transplant recipients from donors aged less than 60 years and donors older than 60 years. **d** Comparison of 1-year graft loss between renal transplant recipients from donors aged less than 50 years and donors older than 50 years. **e** Comparison of 1-year graft loss between renal transplant recipients from donors aged less than 60 years and donors older than 60 years. **f** Comparison of acute rejection (AR) incidence between renal transplant recipients from donors aged less than 50 years and donors older than 50 years. **g** Comparison of acute rejection (AR) incidence between renal transplant recipients from donors aged less than 60 years and donors older than 60 years. **h** Comparison of 1-year post-transplantation eGFR between renal transplant recipients from donors aged less than 50 years and donors older than 50 years. **i** Comparison of 1-year post-transplantation eGFR between renal transplant recipients from donors aged less than 60 years and donors older than 60 years. **l** Comparison of 1-year post-transplantation serum creatinine between renal transplant recipients from donors aged less than 50 years and donors older than 50 years. **m** Comparison of 1-year post-transplantation serum creatinine between renal transplant recipients from donors aged less than 60 years and donors older than 60 years. **n** Comparison of DGF incidence between recipients of grafts from donors aged < 60 and donors aged > 60 years

Six studies reported the effect of donor age and recipient survival [14–19]; Grekas et al. [14], Johnson et al. [15] and Guo et al. [16] compared recipient survival from donors aged above/below 50 years (Fig. 2a).

One-year recipient survival from donors aged over/under 60 years was reported by Grekas et al. [14], Giessing et al. [17] and Beradinelli et al. [18] (Fig. 2b), with no significant difference (p = 0.32) being reported for the 3-year survival either (Fig. 2c).

### Effect of donor age on graft survival

Six studies reported the effect of donor age and graft survival [14–16, 20–22], finding no significant difference in 1-year graft survival between recipients of grafts from donors under/over 50 years of age (Fig. 2d).

Eleven studies compared 1-year graft survival between renal transplant recipients of donors aged under/over 60 years [17, 18, 20, 23–30], finding no significant difference (Fig. 2e).

Kumar et al. [20], De La Vega et al. [21], [21] and Jain et al. [31] compared the incidence of AR between recipients of a graft from donors younger than 50 years and donors older than 50 years. The analysis found no statistical difference between the two groups (Fig. 2f).

Four studies [17, 20, 25, 32] looked at AR incidence between graft recipients from < 60 year-old or > 60 year-old LDs. The analysis found that recipients of renal grafts from donors aged < 60 have a 38% lower risk of developing acute rejection compared to recipients of renal grafts from donors aged > 60 years (p = 0.0004) (Fig. 2g).

Four studies [14, 20, 31, 33] reported 1-year post-transplantation eGFR in recipients of renal grafts from donors aged < 50 and donors aged > 50 years (Fig. 2h). Medium effect size (0.46 95% CI: 0.24–0.67) was seen between the eGFR means of recipients of renal grafts from donors aged < 50 and recipients of renal grafts from donors > 50, and this finding was statistically significant (p < 0.0001).

Three studies[14, 20, 34] compared 1-year post-transplantation eGFR in recipients of renal grafts from donors aged younger than 60 and older than 60 years. Large size effect (1.09 95% CI: − 0.4 to 2.59) was seen between eGFR means of recipients of renal grafts from donors aged < 60 and recipients of renal grafts from donors aged > 60, however this finding was not statistically significant (p = 0.15) (Fig. 2i).

Four studies[15, 16, 20, 35] compared 1-year serum creatinine in recipients of renal grafts from donors aged < 50 and donors aged > 50 years, finding the former on average 0.14 mg/dl lower than that of recipients of donors aged > 50 (p = 0.003) (Fig. 2l).

Three studies [17, 19, 20] compared 1-year post-transplantation serum creatinine in recipients of renal grafts from donors aged < 60 and donors aged > 60 years: again the former was on average 0.24 mg/dl lower than that of recipients of donors aged > 60 (p = 0.01) (Fig. 2m).

In the two studies by De La Vega et al. [21] and Duchenne et al. [22], when comparing serum creatinine between recipients who received a renal graft from a donor aged < 50 and recipients who received a renal graft from a donor aged > 50, no significant difference was found (*p* = 0.25).

Three studies [18, 24, 26] compared DGF incidence between recipients of grafts from donors aged < 60 and donors aged > 60. The analysis found recipients of grafts from donors aged < 60 to be 72% less likely to develop DGF compared to recipients of grafts from donors aged > 60 (p = 0.03), (Fig. 2n). No significant difference was found between the incidence of primary non function between the recipients who received a graft from donors aged > 60 or < 60 (p = 0.88) in the two studies [18, 27] reporting on this outcome from donors aged < 60 and recipients of donors aged > 60.

Table 1 summarizes the evidence of proteinuria in living donor grafts stratified according to age, with no significant difference among grafts from under/over 50 years as well as in the comparison under/over 60 years.

**Table 1:** Effect of donor age on the development of proteinuria in renal transplant recipients

Proteinuria	Proteinuria measurement	Donor age						Statistical significance
		Age < 50	50–55	55–60	60–65	65–69	Age > 70	
Johnson et al. [15]	Proteinuria was measured on postoperative day 1,7,30,90,180,365 and 730. Proteinuria was defined as significant if spot analysis demonstrated > 100 mg of protein in urine on at least 2 occasions	Proteinuria: 21/56 (37.5%)	Proteinuria: 9/22 (40.1%)					P = 0.49 (Chi squared)
		Significant proteinuria: 10/56 (17.9%)	Significant proteinuria: 4/22(18.2%)					P = 0.6 (Chi squared)
Grekas et al. [14]	Proteinuria was measured g/24 h 1 year and 2 years after transplantation	1-year post-transplantation: (N = 12); A: 0.5 ± 0.3	1-year post-transplantation: (N = 23): 0.3 ± 0.1			1-year post-transplantation: (n = 25) 0.3 + -/0.1		No statistical difference was found between protein excretion between the 3 groups at 1 and 2 years
		2-year post-transplantation: (N = 12) 0.3 ± 0.2	2-year post-transplantation: (N = 23): 0.3 ± 0.2,			2-year post-transplantation: (n = 25) 0.3 ± 0.1		

### Effect of donor sex on graft survival

Two studies [36, 37] compared non-death censored graft survival between recipients of grafts from male and female donors. Only Jacobs et al. [36] found recipients of grafts from male donors to have a significantly higher rate of graft survival (89.5%) compared to recipients of grafts from female donors (83%) at 3 years post-transplantation (p = 0.01), as shown in Table 2. Neither study found any significant difference in recipient graft survival between recipients of grafts from donors of the same and opposite sex (Table 3).

**Table 2:** Effect of donor sex on non-death censored renal graft survival in recipient

Study name	Recipient graft survival	Donor sex, recipient graft survival		Chi squared test
		Male	Female	
Jacobs [30]	1-year graft survival	297/313	391/417	p = 0.52
	3-year graft survival	280/313	346/417	p = 0.01
Wafa [31]	5-year graft survival	154/180	74/93	p = 0.21
	10-year graft survival	103/180	60/93	p = 0.24

**Table 3:** Effect of matching sex between donor and transplant recipient on non-death censored graft survival

Study name	Recipient graft survival	Same sex	Different sex	Chi squared test
Jacobs[30]	1-year graft survival	323/339	365/391	P = 0.26
	3-year graft survival	295/339	331/391	P = 0.36
Wafa [31]	5-year graft survival	97/120	131/153	P = 0.28
	10-year graft survival	72/120	91/153	P = 0.93

Jacobs et al. [36] found no difference in 1-year graft survival in male recipients from male donors and female donors (p = 0.15). However, at 3-year follow-up, male recipients of grafts from male donors were found to have higher graft survival (93.2%) compared to male recipients of grafts from female donors (84.1%) (p = 0.006). On the other hand, Wafa et al. [37] found no significant difference in male graft survival from male and female donors both at 5-year [p = 0.97] and 10-year [p = 0.31] post-renal transplantation (Table 4). Neither study found any significant difference in female recipient graft survival from male and female donors. This finding was seen in the studies at both shorter and longer periods after renal transplantation (Table 5).

**Table 4:** Non-death censored graft survival in male renal transplant recipients based on the gender of their donor

Study name	Recipient graft survival	Male to male	Female to male	Chi squared test
Jacobs [30]	1-year graft survival	157/162	225/240	P = 0.15
	3-year graft survival	151/162	202/240	P = 0.006
Wafa [31]	5-year graft survival	47/55	24/28	P = 0.97
	10-year graft survival	31/55	19/28	P = 0.31

**Table 5:** Non-death censored graft survival in female renal transplant recipients based on the gender of their donor

Study name	Recipient graft survival	Male to female	Female to female	Chi squared test
Jacobs [30]	1-year graft survival	140/151	166/177	P = 0.7
	3-year graft survival	129/151	144/177	P = 0.32
Wafa [31]	5-year graft survival	107/125	50/65	P = 0.13
	10-year graft survival	72/125	41/65	P = 0.47

### Effect of donor sex on renal function

**Fig. 3:**
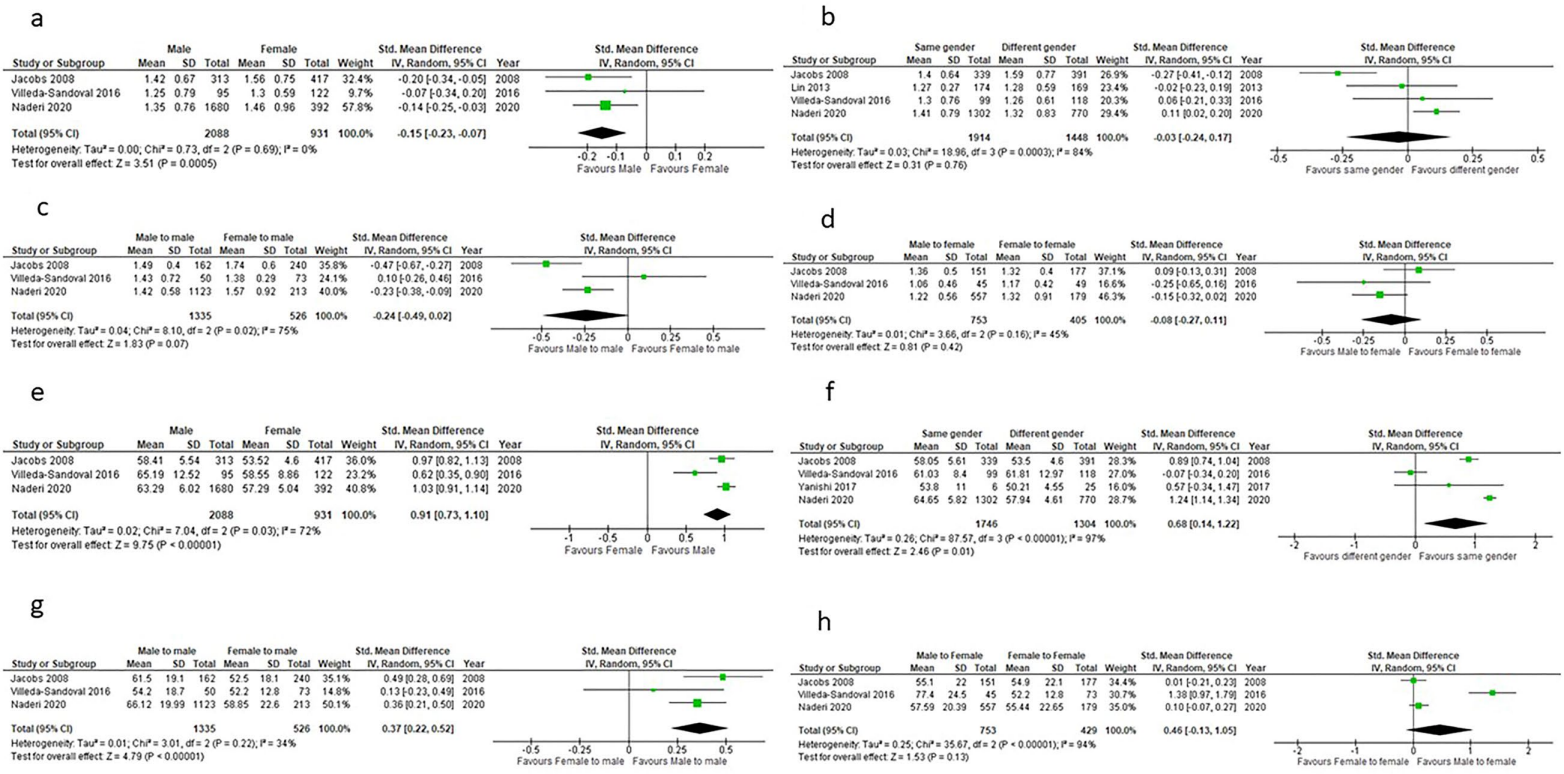
a Effect of donor sex on 1-year post-transplantation serum creatinine in renal transplant recipients. **b** Effect of matching sex between donor and recipient on 1-year post-transplantation serum creatinine in renal transplant recipients. **c** 1-year post-transplantation serum creatinine in male kidney transplant recipients from male and female donors. **d** 1-year post-transplantation serum creatinine in female kidney transplant recipients from male and female donors. **e** Effect of donor sex on 1-year post-transplantation eGFR in renal transplant recipients. **f** Effect of matching sex between donor and recipient on 1-year post-transplantation eGFR in renal transplant recipients. **g** 1-year post-transplantation eGFR in male kidney transplant recipients from male and female donors. **h** 1-year post-transplantation eGFR in female kidney transplant recipients from male and female donors

Five studies [35, 36, 38–40] investigating the effects of donor gender on recipient eGFR or serum creatinine met the inclusion criteria. Overall, recipients of grafts from male donors had 0.12 mg/dl lower serum creatinine compared to recipients from female donors (p = 0.0005), (Fig. 3a).

No significant difference in 1-year post-transplantation serum creatinine was found between recipients of renal grafts from same sex and opposite sex donors (p = 0.78), (Fig. 3b).

The analysis found male recipients from male donors to have, on average, 0.14 mg/dl lower serum creatinine at 1-year post-transplantation compared to male recipients from female donors. However, this finding was not-statistically significant (p = 0.07), (Fig. 3c).

No significant difference was found in serum creatinine levels of female recipients of grafts from male and female donors (p = 0.42), (Fig. 3d).

With regard to the effect of LD sex on eGFR post-transplantation, recipients of grafts from male donors had a significantly higher eGFR compared to recipients of female donors (p < 0.00001), with a large size effect seen between recipients of grafts from male and female donors (0.91 95% CI: 0.73–1.10), (Fig. 3e).

A large effect size was also seen between recipients of grafts from same-sex donors and opposite sex donors (0.68 95% CI: 0.14–1.22). This finding was statistically significant (p = 0.01) where recipients of grafts from same sex donors had a higher eGFR compared to recipients of grafts from opposite sex donors, unlike the difference in serum creatinine between recipients of transplants from same sex and opposite sex donors (Fig. 3f).

Small size effect was seen between the eGFR of male recipients who received their graft from a male and a female donor (0.37 95% CI: 0.26–0.49): male recipients who received their graft from a male donor had a statistically higher eGFR compared to male recipients who received their graft from a female donor (p < 0.00001) (Fig. 3g), while no significant difference was seen in 1-year post-transplantation eGFR between female recipients of grafts from male donors and female donors (p = 0.13), (Fig. 3h).

With regard to the effect of sex matching between recipients of renal transplant from LDs on the development of proteinuria in recipients, results are summarized in Table 6; only Yanishi et al. [40] found proteinuria to be significantly lower in female recipients who had received a graft from a male donor compared to recipients who had received a transplant from a donor of the same gender and to male recipients who had received a renal graft from a female donor.

**Table 6:** Effect of matching recipient’s sex with the sex of their donor on the post-transplantation proteinuria

Study	Proteinuria measurement	Male to Male	Male to Female	Female to female	Female to male	Outcomes reported in the paper
Oh et al. [35]	Proteinuria measured 24 h post-surgery in mg/day	MM (n = 65): 23.4 ± 61.6	MF (n = 34): 81.9 ± 354.4	FF (= 29): 9.7 ± 51.6	FM (n = 67): 36.1 ± 123.8,	Independent sample t-test: MM-FM (p = 0.461), MF-FF (p = 0.282); MM-MF (p = 0.198), FM-FF: (p = 0.273)
Yanishi [34]	Proteinuria measured 1-year post-surgery in mg/day	Group 1(same gender) n = 6: 135.2 ± 98.1	group 2: (male donor to female recipient) (n = 8). 63.7 ± 28.7	Group 1(same gender) n = 6: 135.2 ± 98.1	Group 3: female donor to male recipient (n = 17): 205.5 ± 35.2	ANOVA between the 3 groups found the lowest proteinuria to be in the Male to Female group (p < 0.01)

### Effect of donor BMI on recipient outcomes

**Fig. 4:**
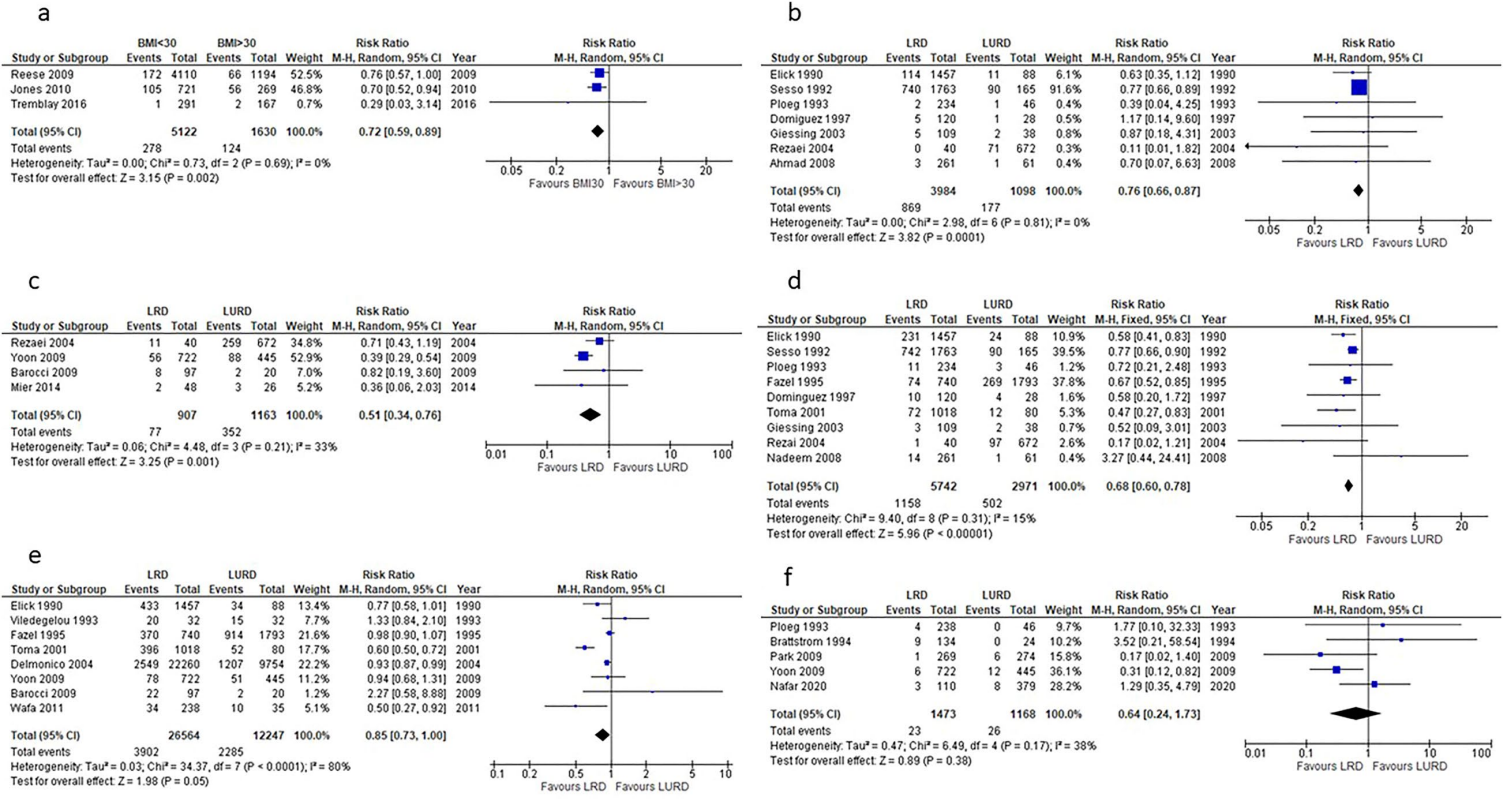
**a** Effect of donor BMI on the post-transplantation incidence of DGF in the recipients. **b** Effect of relationship between recipient and donor on recipient 1-year survival. **c** Effect of relationship between recipient and donor on recipient 10-year survival. **d** Effect of genetic relationship between donor and recipient on recipient 1-year graft survival. **e** Effect of genetic relationship between donor and recipient on 5-year graft survival. **f** Effect of genetic relationship between donor and recipient on graft DGF

Three studies [41–43] compared DGF incidence in recipients of grafts from non-obese (BMI < 30) and obese donors (BMI > 30). Recipients of grafts from non-obese donors had a 27% lower risk of developing DGF compared to recipients of grafts from obese donors(p = 0.002), (Fig. 4a). Two studies [41, 44] compared the incidence of AR between recipients of renal grafts from donors with BMI < 30 and BMI > 30, with no overall significant difference (p = 0.25).

### Effect of relationship between donor and recipient on outcomes in recipients

Ten studies [17, 45–53] looked at the effect of the relationship between donor and recipient outcomes. In Figs. 4b, c, recipient survival at 1-year and 10 years is favored by a biological relationship (p < 0.0001).

The same beneficial effect of a genetic relationship between donor and recipient is noted on 1-year and 5-year graft survival (Figs. 4d, e).

In terms of graft function, with a mean follow-up of 45 months, Ahmad et al. [45] noted that eGFR was 59 ± 29 in biologically related LDs versus 49 ± 14 ml/min/1.73 m^2^ in living unrelated donors (LURDs). Similar findings for serum creatinine were reported by Giessing et al. [17] and Brattstrom et al. [54]

Along the same line, even DGF incidence favored genetically related LDs (Fig. 4f), although not significantly, while contrasting results are reported on the incidence of AR.

## Discussion

Kidney transplant survival severely hinders the quality of the implanted graft, with living donation offering numerous advantages on recipient outcomes due to a better intrinsic quality of the implanted organ and the lower susceptibility to ischemic reperfusion injury [55]. In the present review, we looked at the evidence of how the demographic factors of age, sex, BMI and genetic relationship with the recipient influence post-transplant survival, graft function and acute rejection. These findings need consideration for guidance on donor-recipient matching, with particular regard to the implementation of sharing schemes, including poorly matched couples, thus providing new possibilities for prospective couples.

With regard to age, our analysis found that recipients of grafts from LDs aged < 60 have a 38% lower risk of developing acute rejection compared to those aged > 60 years. This result leads to the open debate on immunosuppression in the elderly, in whom, although physiological immunosenescence linked to biological aging is known, other potential contributors, such as the engraftment of older organs, is associated with higher rejection rates, and thus the need for tailored, age-adopted immunosuppression [56].

Additionally, this finding might also be the consequence of a more distant biological relationship in aged couples, where the donation is usually between spouses, as opposed to younger ones, where instead the donation happens more often between related subjects [25].

Furthermore, recipients of grafts from donors aged < 60 are 72% less likely to develop DGF compared to recipients of grafts from donors aged > 60 (p = 0.03), in agreement with previous reports on the link between DGF and acute rejection [57]. An interesting finding is that proteinuria in recipients of LD grafts stratified according to age, shows no significant difference between older and younger donors, highlighting that the intrinsic quality, i.e. the podocyte barrier, is still high as LDs are healthy, screened individuals.

In the present meta-analysis, as in the case of standard donor criteria, we found superior one-year eGFR in recipients of grafts from donors younger than 50 years, compared to those older than 50 years; this effect was not confirmed when using 60 years as a cut-off, although in the latter case we noted a potentially large effect size (1.09 95%CI: -0.4 to 2.59) and a smaller number of included studies (three versus four). A complete discussion of the effect size for each of the parameters considered is presented in Appendix 3 in ESM.

With regard to the effect of LD sex on eGFR post-transplantation, recipients of grafts from male donors were found to have a significantly higher eGFR compared to recipients of female donors (p < 0.00001). This might be linked to a nephron mass effect [58], but it is controversial whether other possible factors could be concurring, considering the higher incidence of chronic kidney disease in women.

Looking at donor BMI, recipients of grafts from non-obese donors had a 27% lower risk of developing DGF compared to recipients of grafts from obese donors (p = 0.002), while regarding the incidence of acute rejection, no overall significant difference in acute rejection was observed.

Finally, concerning the genetic relationship with the recipient, graft function and survival were favored by the biological link between donor and recipient, possibly in relation of better histocompatibility [59], that also reduces the incidence of acute rejection, as previously discussed. Given that some transplant candidates may have multiple potential donors to choose from, a better understanding of the association between donor-recipient biological relationship and post-transplant outcomes can improve donor selection. Notably, living donation among elderly subjects (> 60 years) almost always occurs between unrelated recipients and our data show that,therefore, also recipient factors, such as older age might influence graft survival.

## Limitations

The retrospective nature of the analyzed studies limits the level of evidence we were able to achieve, based on observational registry data, a small number of studies and considerable heterogeneity.

## Conclusion

In conclusion, the age of LDs is likely to impact on recipient outcomes. Donor BMI affects DGF incidence, and recipients of genetically related and male donors have better 1-year eGFR and graft survival. Future larger studies are warranted to identify the optimal donor-recipient matching and to guide towards establishing living donor exchange programs, even internationally, or involving compatible pairs, in order to generate more exchange opportunities and achieve better results.

## Supplementary Information

Below is the link to the electronic supplementary material.

Supplementary file1 (DOCX 17 kb)

Supplementary file2 (DOCX 27 kb)

Supplementary file3 (DOCX 13 kb)

## Abbreviations

AR: Acute rejection
BMI: Body mass index
DGF: Delayed graft function
LD: Living donor
LKD: Living kidney donation
PNF: Primary non function
