# Supplementary material for: How good is a living donor? Systematic review and meta-analysis of the effect of donor demographics on post kidney transplant outcomes
Source: J Nephrol. 2022 Jan 24;35(3):807–20. doi: 10.1007/s40620-021-01231-7 (PMC8995249; doi:10.1007/s40620-021-01231-7)
Supplement: Supplementary file 1 — Supplementary file1 (DOCX 17 kb) [file 40620_2021_1231_MOESM1_ESM.docx]

**Appendix 1. Search strategy**

EMBASE and MEDLINE databases were searched through Ovid on 14/11/2020, the search algorithm used is shown in table 1a. English language filter was applied to the search.

**Table 1a. Search algorithm used to search EMBASE and MEDLINE databases through Ovid.**

| Step | Input |
| --- | --- |
| 1 | gender/ or "gender and sex"/ |
| 2 | sex/ or sex difference/ |
| 3 | sex |
| 4 | age/ |
| 5 | ethnicit* |
| 6 | ethnic minorit* |
| 7 | BAME |
| 8 | exp "ethnic or racial aspects"/ |
| 9 | BMI/ |
| 10 | BMI or weight |
| 11 | genetic relationship/ |
| 12 | 1 or 2 or 3 or 4 |
| 13 | 5 or 6 or 7 or 8 or 9 or 10 or 11 |
| 14 | 12 and 13 |
| 15 | exp kidney donor/ |
| 16 | kidney transplantation/ |
| 17 | living donor/ |
| 18 | exp graft recipient/ |
| 19 | 15 or 16 or 17 or 18 |
| 20 | 14 and 19 |

Web of Science core collection, BIOSIS (1950-2008), CABI, Korean Journal database, Russian Science Citation Index and SciELO were searched through Web of Science search engine on 14/11/2020. The search algorithm used is shown in table 1b.

**Table 1b. Search algorithm used to search Web of Science core collection, BIOSIS (1950-2008), CABI, Korean Journal database, Russian Science Citation Index and SciELO through Web of Science.**

| Step | Input |
| --- | --- |
| 1 | TS=(sex or gender) |
| 2 | TS=(sex and difference) |
| 3 | TS=age |
| 4 | TS=(ethnicit* or ethnic minorit*) |
| 5 | TS=BAME |
| 6 | TS=(ethnic* or race) |
| 7 | TS=(BMI or weight) |
| 8 | TS=genetic relationship |
| 9 | #1 or #2 or #3 |
| 10 | #4 or #5 or #6 or #7 or #8 |
| 11 | #9 and #10 |
| 12 | TS=kidney |
| 13 | TS=transplantation |
| 14 | TS=(living or live or non-deceased) |
| 15 | TS=(donor) |
| 16 | TS=graft |
| 17 | TS=recipient |
| 18 | #12 and #13 and #14 and #15 and #16 and #17 |
| 19 | #11 and #18 |

Cochrane library database was searched on 14/11/2020. The search algorithm used is shown in table 1c.

**Table 1c. Search algorithm used to search the Cochrane library database**

| Step | Input |
| --- | --- |
| 1 | MeSH descriptor: [Gender Identity] this term only |
| 2 | MeSH descriptor: [Sex] this term only |
| 3 | MeSH descriptor: [Sex Characteristics] this term only |
| 4 | (sex):ti,ab,kw |
| 5 | MeSH descriptor: [Age Factors] this term only |
| 6 | ethnicit* |
| 7 | ethnic minorit* |
| 8 | BAME |
| 9 | BMI |
| 10 | weight |
| 11 | MeSH descriptor: [Family] explode all trees |
| 12 | genetic relationship |
| 13 | MeSH descriptor: [Ethnic Groups] explode all trees |
| 14 | MeSH descriptor: [Continental Population Groups] explode all trees |
| 15 | #1 or #2 or #3 or #4 or #5 |
| 16 | #6 or #7 or #8 or #9 or #10 or #11 or #12 or #13 or #14 |
| 17 | #15 or #16 |
| 18 | MeSH descriptor: [Kidney] explode all trees |
| 19 | MeSH descriptor: [Tissue Donors] explode all trees |
| 20 | MeSH descriptor: [Transplantation] explode all trees |
| 21 | MeSH descriptor: [Transplant Recipients] explode all trees |
| 22 | #18 and #19 |
| 23 | #18 and #20 |
| 24 | #18 and #21 |
| 25 | #22 or #23 or #24 |
| 26 | Kidney 51158 |
| 27 | donor or transplantation or recipient or transplant |
| 28 | #26 and #27 |
| 29 | #17 AND #25 |
| 30 | #17 AND #28 |
| 31 | #29 or #30 |
