# Supplementary material for: How good is a living donor? Systematic review and meta-analysis of the effect of donor demographics on post kidney transplant outcomes
Source: J Nephrol. 2022 Jan 24;35(3):807–20. doi: 10.1007/s40620-021-01231-7 (PMC8995249; doi:10.1007/s40620-021-01231-7)
Supplement: Supplementary file 3 — Supplementary file3 (DOCX 13 kb) [file 40620_2021_1231_MOESM3_ESM.docx]

**Appendix 3: The role of effect size**

Effect sizes were discussed for figures 2h, 2i and 3a-3h in the main text. Cohen’s d was used to calculate the effect size. Z score of <0.2 was considered to be small effect size. Z score of 0.2-0.8 was considered to be a moderate effect size. Z score of >0.8 was considered to be a large effect size.

Figure 2a: Moderate effect size (Z=0.46), however there was no significant difference between recipients of donors age<50 and donors age>50 (p=0.65).

Figure 2b: Moderate effect size (Z= 0.39), however there was no significant difference between recipients of donors age<60 and donors age>60 (p=0.69).

Figure 2c: Large effect size (Z=1.01) was seen, however no significant difference was seen between the recipients of donors aged<60 and aged>60 (p=0.31).

Figure 2d: Large effect size (Z=1.25), however there was no significant difference between recipients of donors aged<50 and donors aged>50 (p=0.21).

Figure 2e: Large effect size (Z=2.37) was seen. Recipients of grafts from donors aged<60 had a significantly better 1 year renal graft survival compared to recipients of grafts from donors aged>60 (RR: 0.55, 95%CI: 0.34 -0.9; p=0.02).

Figure 2f: Large effect size (Z=2.14) was seen. Recipients of grafts from donors aged<50 had a significantly lower rate of acute rejection compared to recipients of grafts from donors aged>50 (RR: 0.70, 95%CI: 0.51 to 0.97; p=0.03).

Figure 2g: Large effect size was seen (Z= 3.54). Recipients of grafts from donors aged<60 had a significantly lower incidence of acute rejection compared to recipients of grafts from donors aged>60 (RR: 0.63, 95%CI: 0.49 to 0.81; p=0.0004).

Figure 2h: Large effect size was seen (Z= 4.18). 1 year post-transplantation eGFR was significantly higher in recipients of donors age<50 compared to recipients of donors >50 (standardized mean difference: 0.46, 95%CI: 0.24 to 0.67; p<0.0001).

Figure 2i: Large effect size was seen (Z= 1.43) however no significant difference was seen between the two groups (p=0.15).

Figure 2l: Large effect size was seen (Z= 2.94). Recipients of grafts from donors aged<50 had a significantly higher 1 year post-transplantation serum creatinine compared to recipients of grafts from donors aged>50 (mean difference: -0.14, 95%CI: -0.23 to -0.05; p=0.003).

Figure 2m: Three studies [11, 13, 14] compared 1-year post-transplantation serum creatinine in recipients of renal grafts from donors aged <60 and donors aged >60 years. Serum creatine in recipients of renal grafts from donors aged <60 : again the first was on average 0.24 mg/dl lower than of recipients of grafts from donors aged>60 (p=0.01). Large effect size was seen (Z= 2.46).

Figure 2n: Large effect size (Z= 2.17), recipients of grafts from donors aged<60 had a significantly lower rate of DGF compared to recipients of grafts from donors aged>60 (RR: 0.29 95%CI: 0.09 to 0.89; p=0.03).

Figure 3a: Large effect size (Z= 3.51), recipients of grafts from male donors had a significantly lower 1 year post-transplantation serum creatinine compared to recipients of grafts from female donors (Standardised mean difference: -0.15, 95%CI: -0.23 to -0.07; p=0.0005).

Figure 3b: Medium effect size (Z=0.31) was seen. There was no significant difference in 1 year post-transplantation in serum creatinine between recipients of grafts from same gender and different gender donors (p=0.76).

Figure 3c: Large effect size was seen (Z=1.83). However there was no significant difference in 1 year post-transplantation serum creatinine between male recipients of kidney grafts from male and female donors (p=0.07).

Figure 3d: Medium effect was seen (Z=0.81). However there was no significant difference in 1 year post-transplantation serum creatinine between female recipients of kidney grafts from male and female donors (p=0.42).

Figure 3e: Large effect size was seen (Z=9.75). Recipients of grafts from male donors had a significantly higher eGFR 1 year post-transplantation compared to recipients of grafts from female donors (Standardised mean difference: 0.91, 95%CI: 0.73 to 1.10) (p<0.00001).

Figure 3f: Large effect size was seen (Z=2.46). Recipients of grafts from the same sex had a significantly higher 1 year post-transplantation eGFR (Standardised mean difference 0.68, 95%CI: 0.14 to 1.22) (p=0.01).

Figure 3g: Large effect size was seen Z=4.79. Male recipients of grafts from male donors had a significantly higher 1 year post-transplantation eGFR compared to male recipients of grafts from female donors (Standardised mean difference: 0.37, 95%CI: 0.22 to 0.52) (p<0.00001).

Figure 3h: Large effect size was seen (Z=1.53). There was no significant difference in 1 year post-transplantation eGFR between female recipients of renal grafts from male and female donors (p=0.13).

Figure 4a: The incidence of DGF was significantly lower in recipients of renal grafts from donors with BMI<30 compared to recipients of grafts from donors BMI>40 (RR: 0.72, 95%CI: 0.59 to 0.89) p=0.002. The effect size was large Z=3.15.

Figure 4b: Recipient survival at 1 year post-transplantation was significantly higher in recipients of grafts from living related donors compared to recipients of grafts from living unrelated donors (RR: 0.76, 95%CI: 0.66 to 0.87; p=0.00001). The effect size was large (Z=3.82).

Figure 4c: Recipient survival at 10 years post-transplantation was significantly higher in recipients of grafts from living related donors compared to recipients of grafts from living unrelated donors (RR: 0.51 95%CI: 0.34 to 0.76; p=0.001). The effect size was large (Z=3.25).

Figure 4d: Recipient graft survival at 1 year post-transplantation was significantly higher in recipients of grafts from living related donors compared to recipients of grafts from living unrelated donors (RR: 0.68 95%CI: 0.60 to 0.78; p<0.00001). The effect size was large (Z=5.96).

Figure 4e: Recipient graft survival at 5 years post-transplantation was not significantly different in recipients of grafts from living related donors compared to recipients of grafts from living unrelated donors (RR: 0.85 95%CI: 0.73 to 1.00; p=0.05). The effect size was large (Z=1.98)

Figure 4f: Delayed graft function was not significantly different between the recipients of grafts from living related donors and living unrelated donors (p=0.38). The effect size was large (Z=0.89).
